# Determinants of aged cheese consumer preferences in Morocco—a cross-sectional study of economic, cultural, and social factors influencing purchasing behaviors

**DOI:** 10.3389/fnut.2025.1600873

**Published:** 2025-07-11

**Authors:** Nouhayla Mouatadid, Mourad Oukheda, Mustapha Khiati, Rachid Saile, Anass Kettani

**Affiliations:** ^1^Laboratory of Biology and Health, URAC 34, Faculty of Sciences Ben M’sik, Health and Biotechnology Research Center, Hassan II University of Casablanca, Casablanca, Morocco; ^2^Royal Institute for the Training of Youth and Sports Executives, Rabat, Morocco; ^3^Department of Communication Sciences and Humanities, Faculty of Sciences Ben M’sik, Hassan II University of Casablanca, Casablanca, Morocco

**Keywords:** aged cheese, authenticity, consumer preferences, local market, consumption frequency, purchasing power, choice factors

## Abstract

The shift in dietary habits, driven by increased exposure to global gastronomy and improved living standards, has led to a rise in the consumption of aged cheese, also known as matured cheese, worldwide. Although considered premium products, these cheeses transcend their culinary status to embody a cultural heritage, even in countries where this culture is not historically rooted, such as Morocco. However, this trend primarily affects consumption, while local production remains limited, reinforcing dependence on imports. In addition to their gastronomic dimension, these products are being increasingly promoted due to a growing awareness of their benefits, which is stimulating greater interest among consumers. This study aims to redefine the status of mature cheeses in Morocco by identifying the factors influencing their acceptability and consumer preferences. An online questionnaire, validated and distributed to a representative sample of the population, was used to statistically analyze these trends. The results reveal a promising market, characterized by strong interest in these products and frequent consumption, influenced by criteria related to the product’s characteristics and economic factors that shape the purchase decision, while the impact of sociodemographic variables remains more moderate. The acceptability of mature cheeses, particularly local products, is accompanied by a willingness on the part of consumers to favor quality over price, as well as a significant attachment to national production, which has been demonstrated. This dynamic opens up favorable prospects for the development of national production, reducing dependence on imports, and offering local producers an opportunity to strengthen their position in a market that remains relatively uncompetitive. A move upmarket, supported by a targeted marketing strategy that takes account of identified consumer disincentives and promotes the “local” character, would be an effective lever for boosting this sector, especially as the results indicate a marked appeal for these products.

## 1 Introduction

The design and commercialization of new food products on a global scale are complex processes, where a multitude of interdependent factors, including esthetic, cultural, and political characteristics, that affect consumer acceptance (1). Cheese consumption continues to grow, particularly in Europe, the world’s leading exporter, ahead of the United States and Oceania. At the same time, innovation in the cheese sector is intensifying to meet the growing expectations of consumers (2).

Europe stands out for its constant innovation in cheese production and marketing, combining diversity, strict compliance with standards, and preservation of authenticity. In 2015, the European Union produced nearly 10 million tons of cheese and accounted for 32% of global exports (3). Each country has a preference for certain types of cheese. For example, European countries are renowned for cheeses such as feta and cheddar, while others have a more varied consumption pattern (1). In Turkey, for example, certain varieties of cheese, such as tulum or Yozgat Çanak, are named and consumed according to their production method, the type of milk used, and the storage conditions, and are associated with the regions from which they originate (4). Therefore, Europe boasts a rich diversity of traditional cheeses, particularly those that emphasize their specific origin, as evidenced by the numerous Protected Designations of Origin (PDOs). Origin labeling thus serves as a key tool for differentiation and promotion, as illustrated by studies conducted in Castilla-La Mancha, where 61.4% of consumers express a preference for local Manchego cheese bearing a Denomination of Origin (DO) (5).

Cheese consumption is now expanding to regions where it was previously limited, driven by effective marketing strategies and the scientific recognition of its nutritional benefits (6). In Africa, fermented dairy products have long been a vital part of the rural diet, despite the historically limited production of mature cheeses (6). Furthermore, although cheese consumption remains higher in specialized countries, this gap is narrowing thanks to population growth, economic dynamism, and changing lifestyles (7). For illustration, the South African dairy industry has seen significant growth in cheese production and consumption, with an 18.5% increase in per capita consumption since 1995. This development has been driven by a multitude of factors, including those mentioned above (8). The varieties of cheese differ depending on the cultural structure of countries, climatic conditions, the diversity of local animals, and production techniques (9). In the Mediterranean region, cheeses made from sheep’s and goat’s milk are distinguished by their flavor and composition, shaped by traditional farming practices and local conditions. Despite the evolution of modern techniques, brined white cheeses, such as Feta, remain predominant due to their deep historical and cultural roots (10), especially as this type of product reflects the identity of a region through the origin of the milk, the pastures, and traditional recipes, thereby influencing consumption practices (11). However, changes in health requirements and processing technologies are redefining the production of Mediterranean cheeses, whose future will depend on striking a balance between tradition and innovation to meet the expectations of developed markets (12). Given that the combination of science and technology has become a key lever for optimizing quality, in the face of growing demand that has necessitated the switch to industrialized production systems (13). In many countries, where cheese production remains limited, imports are the main source of supply (14). Between 2008 and 2012, global cheese exports increased by an average of 4.6% annually in response to rising international demand (15).

Although these data are old, they illustrate the evolution of cheese consumption during a period marked by significant efforts to diversify, improve quality, and stimulate demand. With the global expansion of cheese production, historically importing countries (6). Countries such as Morocco are also experiencing a change. Although this country has no marked cheese-making tradition, it is now benefiting from economic development and changing lifestyles (16), encouraging the adoption of new eating habits under the influence of foreign cultures (17). The consumption of mature cheeses has increased significantly and is now an integral part of the daily diet of Moroccans in various forms and contexts. However, the Moroccan market still relies heavily on imported cheeses due to the challenges faced by the local cheese industry, particularly regarding the acceptance of cheeses, especially locally produced mature cheeses, in a region where the cheese-making tradition is still underdeveloped (14).

However, Morocco, a major importer of aged cheeses, is now seeking to develop local production, particularly through the optimization of camembert manufacturing (18), particularly in a context where production is mainly geared toward fresh cheeses, which are rapidly consumed due to their limited shelf life (19). The information available on the Moroccan cheese sector, particularly regarding consumption and production trends, remains limited and insufficiently studied. This situation highlights the originality of this study, which stands out not only internationally but also specifically in the Moroccan context. It is part of an approach aimed at redefining the cheese culture in Morocco and contributing to the development of mature cheeses in a region where this topic remains largely underexplored.

Adding value to mature cheeses requires an integrated approach, highlighting their unique sensory qualities resulting from the maturing process, as well as their nutritional benefits, particularly their high protein, vitamin, and calcium content, which are essential for a balanced diet (20). Attractive packaging and quality labels can be used to add value, guaranteeing authenticity and excellence (21). These initiatives are particularly relevant, feasible, and accessible in regions such as Morocco, where the cheese-making culture is not yet deeply rooted. They promote and protect products, benefiting both producers and consumers. This model, already applied in contexts aiming to replicate the characteristics of imported products, is part of the European Union’s food safety policy, particularly through labeling (22). In this respect, producing quality cheese is not enough; substantial marketing investment is essential to build a competitive reputation and strengthen the manufacturer’s brand image in the face of producers from specialized countries (23). Furthermore, a high-quality product is even more likely to succeed in the market when it is considered a heritage item, carrying a rich culinary legacy. The marketing of cheese can be based on a specific regional tradition (24), reinforced by the acquisition of labels and certifications that enhance authenticity, geographical origin, and traditional production methods. These elements, deeply rooted in local history and traditional expertise, enable producers to differentiate themselves from established brands, which often rely on identity narratives and collective labels such as Geographical Indications (GIs), a widely recognized form of recognition that is effectively communicated to stakeholders (25). This enables the product to be positioned in more lucrative market segments, standing out in a saturated environment while appealing to a clientele that values quality and authenticity. Nevertheless, this sector remains vulnerable to economic constraints, as production, particularly of mature cheeses, remains lengthy, costly, and unprofitable, necessitating high selling prices. This reality can limit the accessibility of the product, particularly in regions where cheese-making culture is not yet deeply rooted, such as in Morocco, leading to price disparities between local and international markets (26). However, a well-crafted marketing strategy can overcome this barrier by appealing not only to local consumers but also to tourists interested in gastronomy and cultural heritage, thus making a significant contribution to the local economy (27). By capitalizing on the evolution of eating habits, there is a growing interest in aged cheeses, alongside the global expansion of cheese culture. This dynamic highlights the ever-increasing recognition of the nutritional and organoleptic qualities of mature cheeses. However, the absence of comprehensive data on this sector in Morocco reveals a potential lag in the systematic development of the industry at the national level. Consequently, it is essential to promote these products by increasing consumer awareness and tailoring them to local preferences. This will help position them as quality benchmarks in both national and international markets, while facilitating their adoption in regions where cheese-making traditions have yet to become deeply ingrained.

This study aims to conduct an in-depth assessment of Moroccan consumers’ preferences for mature cheeses through a comprehensive survey followed by rigorous statistical analysis. This approach will allow us to identify key trends and factors that influence purchasing decisions, providing valuable insights for the sector. The significance of this study lies in its potential to provide strategic recommendations that enhance the acceptability of mature cheeses, improve the competitiveness of the Moroccan cheese sector, and support its expansion into international markets. This research is vital for adapting products to consumer expectations, thus facilitating their integration into a rapidly evolving market. Against this backdrop, we need to look at several crucial issues affecting the market, consumer behavior, and the characteristics of the “aged cheese” product:

1. First of all, is the Moroccan cheese market conducive to investment in the production of mature cheeses, particularly in a context where world consumption is on the increase, which provides a favorable framework for the logic of supply and demand in terms of consumption? In other words, does this represent a challenge for Moroccan producers, who are obliged to convince consumers to adopt local cheeses in a context characterized by the absence of a cheese culture and limited public awareness, or does it constitute an opportunity for players wishing to invest in this sector, which at the same time is marked by relatively weak local competition?
2. With this in mind, what is the current status of cheese consumption in Morocco as “aged cheese”? What are the determinants that influence the flow of consumption and the choice of purchase? Would Moroccan consumers be willing to pay a premium for a product that is not part of their traditional culture? Would they accept a mature cheese produced locally, or would they prefer a well-known imported cheese?
3. Given that authenticity is the main property often associated with recognized mature cheeses, do Moroccan consumers attach any fundamental importance to this characteristic? What strategies could local producers adopt to stand out from the crowd and position themselves effectively in a market that is perceived as saturated by imported products?

We believe that the answers to these questions will enable countries that are not specialized in cheese production, such as Morocco, to better understand qualification strategies, often established by recognized mature cheese brands, based on the preferences of Moroccan consumers.

## 2 Materials and methods

### 2.1 Study design

The aim of assessing the purchasing habits and preferences of consumers of mature cheeses in Morocco is to understand and analyze the factors influencing purchasing decisions, in a context where the local food culture is evolving toward a greater openness to refined cheese products. Considered both as a nutritious alternative for sports enthusiasts and health-conscious consumers and as a delicacy for food lovers, cheese has been highlighted by nutritional studies as an important source of essential nutrients, including proteins, bioactive peptides, amino acids, lipids, fatty acids, vitamins, and minerals (28).

This cross-sectional study focuses on purchasing behavior, economic and cultural influences, and the impact of local preferences and consumer trends. By identifying the key criteria influencing Moroccan consumers’ choices and their level of acceptability, this study aims to highlight opportunities to expand the mature cheese market in Morocco by capitalizing on emerging preferences and under-exploited consumer segments. The study also aims to identify the cultural and traditional obstacles likely to hinder the adoption of mature cheeses by producers, while proposing strategies for overcoming them. This will enable producers to tailor their products to the preferences of Moroccan consumers and implement targeted marketing and communication initiatives to enhance the appeal of mature cheeses to various market segments.

We have adopted methodological approaches based on the technique of collecting data via a validated questionnaire aimed at a representative Moroccan population. Several methods can be employed to achieve this, such as taste tests, field interviews, which involve organizing sessions to assess preferences and record the results, or collecting feedback via discussion channels. However, a survey involving the design of a questionnaire to be administered to the population is the main method used in this study, aiming to collect data on the various aspects that significantly impact product acceptance and the decision to buy. Given that 80% of studies consider it to be the primary data collection tool, it is particularly favored for data requiring statistical analysis and focusing mainly on consumption and behavior (29).

### 2.2 Methodological approach

This study employs a mixed-methods approach, combining both quantitative and qualitative analyses through a multiple-choice questionnaire to objectively assess Moroccan consumers’ preferences for mature cheeses. Adopting an exploratory descriptive approach, the study analyses the factors influencing consumer choices and the acceptability of local ripened cheeses to understand purchasing behavior and identify opportunities for cheesemakers to develop products tailored to local cultural preferences. The descriptive analysis focuses on mapping Moroccan consumers’ current cheese preferences, providing a foundation for producers looking to adjust their offerings to the local market. Meanwhile, the exploratory component evaluates Moroccan consumers’ openness to local ripened cheeses, exploring their expectations and proposing an innovative strategic model to facilitate the integration of these products into their dietary habits, based on rigorous statistical analysis of the collected data. To achieve the objectives of this study, data were collected through a questionnaire administered to a representative sample of Moroccan consumers. A quantitative approach was prioritized to quantify trends, identify relationships between variables, and generate reliable, generalizable data, which will inform the formulation of actionable recommendations for producers and distributors.

### 2.3 Population and sampling

The survey primarily targets Moroccan consumers of mature cheeses, encompassing a diverse range of profiles in terms of culture, age, region, and socioeconomic status. As the questionnaire is distributed online through social networks and other digital platforms, it focuses on a population with internet access. Inclusion criteria require participants to be residents of Morocco, aged 18 or older, consume mature cheeses, and have internet access. Conversely, individuals living outside Morocco, those who do not consume mature cheeses, or those under 18 years of age are excluded from participation. To ensure data quality, all questions, except one open-ended question for suggestions, were mandatory, allowing for the exclusion of incomplete or unreliable responses. These methods facilitate the exportation of responses to a compatible database, enhancing the reliability and validity of data collection, while improving accessibility for participants and enabling efficient use by researchers (30). This dissemination strategy facilitated participant accessibility and optimized data utilization by researchers. It also enabled the reaching of a large, connected audience, open to diverse cultures, while providing the flexibility of participation, which contributed to a higher response rate. To ensure sufficient statistical power, particularly for hypothesis testing and multivariate analyses, a target sample size of over 360 participants was established, ensuring a margin of error of approximately ±5% and a statistical power exceeding 0.80.

Considering the distribution method and the profile of respondents, the sample may be representative of a specific approach to exploratory research that takes into account the behavior of the population in urban Morocco about the consumption of matured cheeses. A final sample of 510 participants was selected to maintain a low margin of error and high confidence levels, both of which are crucial for identifying significant trends. These sampling methods have been successfully applied in survey contexts (31).

### 2.4 Questionnaire design

The questionnaire was designed using Google Forms by established standards, ensuring that the risk of errors was reduced by paying particular attention to its content, formatting, and methods of use (32). The questionnaire consists of 26 multiple-choice questions and begins with a brief introduction outlining the survey’s objectives. This introduction is designed to raise participants’ awareness of the importance of their responses in guiding the cheese industry toward solutions that align with local preferences. By encouraging informed participation, this approach enhances the reliability of the data collected. The questions are organized into four main categories to structure the analysis of consumer preferences and perceptions. The first category, consisting of questions”Q1–Q4,” focuses on the demographic and socioeconomic profile of participants, including factors such as age, gender, income, and geographic region. Categorical options were provided for these questions, particularly for income, which is divided into five levels. This approach aims to minimize respondents’ reluctance to share sensitive information, such as income, which is often withheld due to confidentiality concerns (31). These data allow us to analyze correlations between sociodemographic characteristics and the consumption of mature cheeses, providing a comprehensive overview of consumer segments in Morocco. The second category, comprising seven questions “Q5 to Q11,” focuses on the purchasing behavior and usage patterns of mature cheeses. It covers aspects such as purchase frequency, preferred points of sale (e.g., supermarkets, local markets, and specialty stores), and culinary uses. These factors are essential for understanding the drivers behind consumers’ purchasing decisions. The third section of the questionnaire, covering questions “Q12–Q18,” addresses consumers’ taste and nutritional preferences, including factors such as texture, flavor, fat content, and desired attributes in ripened cheeses. This section aims to identify the criteria influencing consumers’ choices, as well as their expectations regarding quality and nutritional value. In addition, the last category, also composed of seven questions “Q19–Q25,” explores consumers’ perceptions of the quality and environmental aspects of cheese. This includes their preference for locally produced items and their willingness to pay a premium for organic and eco-friendly products. It also considers their purchasing power and their level of interest in mature cheeses, contributing valuable insights to the literature on this topic. An open-ended question, Q26, was included to collect participants’ suggestions and expectations. Overall, the questionnaire provides a comprehensive analysis of the evolution of cheese culture in Morocco, product acceptability, and evaluates the potential of the Moroccan market for this type of production, both domestically and internationally.

### 2.5 Pretest and validation of the questionnaire

Before the questionnaire was officially distributed, a pretest and validation phase was conducted to ensure the relevance, clarity, and validity of the data collection tool. The questionnaire, including all the items, was evaluated. This evaluation verified the clearness, relevance, and coherence of the items, as well as their capacity to collect reliable and usable data. The pilot study also enabled the identification and correction of any potential ambiguities or biases, thereby ensuring a higher quality of the collected data. This phase is crucial to ensure that the questionnaire aligns with the study’s objectives and provides reliable and usable information (32). To ensure the validity of the questionnaire, it was reviewed by experts in economics and nutrition, who assessed the relevance and appropriateness of the questions. Initially, the questionnaire was validated by experts in the field, specifically an economist and professor at the stands for Faculty of Sciences Ben M’Sik, Hassan II University (FSBM UH2) Casablanca, as well as a researcher in economics, management, and international trade. In addition, a biochemist-nutritionist, a research professor at the stands for Biology and Health Laboratory, Faculty of Sciences Ben M’Sik, Hassan II University (LBS FSBM UH2), helped optimize the questions related to the preferences and nutritional aspects of cheeses, ensuring they reflected consumers’ selection criteria, such as nutritional quality and specific dietary needs. This professional insight also ensured that the wording of the questions was appropriate and understandable for respondents, while aligning with current trends in nutrition and health.

The economist, for his part, examined aspects related to purchasing behavior and analyzed the needs of the Moroccan market. His expertise was essential in adapting the questionnaire to local economic circumstances, enabling us to assess the advisability of introducing the production of mature cheeses in Morocco and to explore consumers’ economic and social perceptions of local products. This validation by an economist helps ensure that the collected data offers a perspective that is both relevant and usable for concrete applications in the Moroccan market. Based on their feedback, adjustments were made to enhance question clarity and accuracy. Simultaneously, a pilot test was conducted with a small sample of approximately 40–60 participants to assess the clarity of the questions, identify potential ambiguities, and measure the time required to complete the questionnaire. This pilot study, conducted with a group of 60 participants, representing different sociodemographic categories. This preliminary phase provided an opportunity to gather feedback on the comprehensibility of the questions, the time required to complete the questionnaire, and the interest shown by participants in the various aspects covered, as well as to gain an insight into how the results would be used. The feedback was used to refine certain questions and reorganize the questionnaire’s structure to improve its flow and coherence. Additionally, this pretest was used to assess the feasibility of the survey, specifically by ensuring that participants understood the questions asked and answered them accurately and completely. After implementing the modifications based on the pilot test results, the questionnaire was finalized, ensuring its reliability for the main data collection process.

### 2.6 Data collection

The questionnaire was distributed via Google Forms, a secure online platform accessible through a simple link, enabling us to reach a broad population across Morocco. The online interface was user-friendly, allowing participants to complete the survey in 5–10 min, which helped minimize the risk of disengagement during the process. Each post on social networks included a clear and engaging message, briefly explaining the study’s objectives and the potential impact of participants’ responses on the Moroccan cheese sector. Support was available to address any questions and clarify any aspects as needed. Additionally, an explanatory box outlined the ethical considerations of the research, ensuring the anonymity and confidentiality of the data, thereby fostering a climate of trust and transparency that encourages honest and informed participation. This approach was chosen due to the widespread use of the Internet, allowing us to target a representative online consumer population. The method is advantageous due to the reduced costs and the increasing popularity of the Internet, which serves as a communication and information access tool across various segments of society, further reinforcing the relevance of this method (33). Internet users likely share similar consumption habits with the general population, meaning that an online respondent may indirectly represent those who do not use the Internet. This approach aligns with modern consumer behavior, which is increasingly influenced by online information. Digital tools allow for better control of data quality, reducing interviewer interaction bias, while the anonymity of the online format encourages more honest and thoughtful responses. Moreover, online data collection is fast, cost-effective, and provides the ability to reach a wide range of Moroccan populations, taking into account their cultural specificities regarding mature cheese consumption. This method also enabled the collection of a large volume of data in a short time, while reducing both material and human costs compared to traditional methods. The growing interest among researchers in using online surveys can be attributed to the relative ease and cost-effectiveness of collecting and utilizing digital data, as opposed to traditional face-to-face interviews. Questionnaire-based studies gather information from participants through online communication technologies, such as e-mail or survey platforms.

### 2.7 Respondent profile

#### 2.7.1 Demographic characteristics

This section presents a descriptive analysis of the demographic characteristics of the participants, focusing on key variables such as age, gender, and place of residence. Respondents’ ages were categorized into the following three relevant generational bands: From 18 to <39 years, from 39 to <64 years, and ≥64 years. The geographical breakdown is based on Morocco’s 12 administrative regions, as established by decree number 2.15.10 of 20 February 2015, which outlines their names. These regions are: Tangier-Tétouan-Al Hoceima, l’Oriental, Fès-Meknès, Rabat-Salé-Kénitra, Béni Mellal-Khénifra, Casablanca-Settat, Marrakech-Safi, Drâa-Tafilalet, Souss-Massa, Guelmim-Oued Noun, Laâyoune-Sakia El Hamra, and Dakhla-Oued Ed-Dahab. These regions were presented in detail, accompanied by a drop-down list indicating the cities that comprise them.

#### 2.7.2 Socioeconomic characteristics

Income was stratified into five distinct levels to reflect a more nuanced financial scale. Income levels were defined as “very low (≤3,000 DH), “low” (from >3,000 DH to ≤5,000 DH), “moderate” (from >5,000 DH to ≤ 8,000 DH), “high” (from >8,000 DH to ≤ 12,000 DH), and “very high” (>12,000 DH). The purpose of this classification is to enable participants to select the option that best represents their financial situation. These variables provide a detailed profile of the consumers surveyed, while incorporating data from studies of the Moroccan middle class, examining the differences between subjective perceptions and monetary approaches (16). This analytical approach is essential for identifying variations in behavior and preferences based on sociodemographic and socioeconomic characteristics. The main objective is to segment the population to highlight the groups most likely to consume mature cheeses. The results of this analysis will be presented in the form of tables and graphs to facilitate the visualization of the main trends and their link with the consumption habits and status of the respondents.

### 2.8 Accessibility and consumption habits

This section analyses the consumption habits of participants, focusing on the frequency of consumption of ripened cheese and perceived accessibility to various outlets. Respondents were categorized based on their consumption frequency: daily, weekly, occasional, and rare. Participants who reported never consuming ripened cheeses were excluded from the study, allowing us to focus on a population that aligns with the research objectives and providing a clearer understanding of their consumption patterns. The section also examines the accessibility of mature cheeses across different outlets, including supermarkets, local grocery stores, farmers’ markets, specialty stores, and online platforms. Online purchasing was included as an option for participants who may not have access to these outlets locally but still search for their preferred products online. An “other” option was also provided to capture additional potential sources of supply. This approach aims to expand the analysis of purchase locations and gain a deeper understanding of participants’ preferences, while also assessing whether product availability influences consumption frequency and identifying potential barriers to regular consumption.

### 2.9 Selection criteria and cheese preferences

This part of the analysis examines consumer preferences for aged cheese and the criteria that influence their choices. The aspects covered include economic factors and cheese preferences. This section contains several multiple-choice questions on various elements that have an impact on the purchasing decision, such as the price of cheese in Morocco, the origin of the raw material, the brand, organic certifications, and labels, which are considered to be attractive to consumers (14). Production conditions are also explored, given that authenticity, a key characteristic desired by consumers (34), is often associated with specific traditional methods (35). Multiple-choice questions allow participants to select several criteria that influence their purchasing behavior. Preferences regarding cheese types within the Moroccan population are analyzed across five main categories, based on a fundamental classification of ripened cheeses (36). Culinary uses, including fondues, hot sandwiches, salads, fresh dishes, and pairings with drinks and fruit also influence participants’ choices. This model captures the nuances of food preferences, providing a detailed view of consumers’ specific expectations. Questions typically offer three to four response options, depending on the significance of the issue, with occasional open-ended responses to gather additional details. This section also evaluates Moroccan consumers’ concern about the environmental impact of the cheese industry, identifying varying levels of ecological sensitivity among participants. The statistical analysis aims to identify consumer priorities, explore correlations between variables, and reveal differences based on sociodemographic profiles. The results will offer valuable insights into consumer expectations and environmental concerns.

### 2.10 Interest in local and organic cheese

This section examines consumer interest in local, sustainable, and organic cheeses, considering their purchasing power and willingness to pay a premium for these products. The analysis begins with preferences regarding the origin of the cheese. Although cheese culture is still evolving in Morocco, the market is primarily dominated by imported products, offering consumers a wide range of well-known brands. This situation highlights a key factor influencing purchasing decisions, often related to preferences for specific brands, as discussed in Section 3. The main objective of this section is, therefore, to assess consumer interest in locally produced mature cheeses. Furthermore, the purchasing power of Moroccan consumers is a key aspect of this study, as mature cheeses are often viewed as luxury items due to their potentially high cost and image (37). In a context where cheese culture is not yet deeply established, these products can be expensive, limiting their widespread adoption. The study examined the relationship between purchasing power and socioeconomic status to differentiate between occasional buyers and regular consumers. Descriptive statistical analysis evaluated respondents’ perceptions of the value of local and organic cheeses, as well as their financial capacity to prioritize these products. The findings highlight opportunities for local producers while also shedding light on the challenges associated with the acceptance of these products in the Moroccan market.

### 2.11 Statistical analysis

The data management was carried out rigorously to ensure its reliability. Responses that did not meet the predefined inclusion criteria were identified and excluded from the analysis. Missing data were handled according to predefined rules, such as removing incomplete questionnaires that contained missing information. Furthermore, outliers were identified through consistency tests and either corrected or excluded as necessary, thereby ensuring the validity and accuracy of the final results. The validated data collected through the questionnaire were analyzed using Statistical Package for the Social Sciences (SPSS) version 27.0 (IBM Corporation). An initial descriptive analysis was performed to summarize the characteristics of the studied variables. This included calculating frequencies for qualitative variables and means and standard deviations for quantitative variables. To assess the normality of the quantitative data, the Kolmogorov–Smirnov test was applied. This test determined whether the observed distributions deviated significantly from a normal distribution, a crucial step in guiding the choice of subsequent statistical tests. Relationships between qualitative variables were examined using the chi-squared (*χ*^2^) test, which identified significant associations between the variables under study. To measure the strength and importance of these associations, Cramer’s V was calculated. This coefficient, which ranges from 0 to 1, provides an estimate of the strength of the relationship between two variables.

A Multiple Correspondence Analysis (MCA) was also conducted to explore the groupings and associations between qualitative variables. This method allowed for a multidimensional visualization of the complex relationships between cheese consumption preferences and other sociodemographic or behavioral variables. MCA offered an overview of patterns and interactions, facilitating the interpretation of results. All analyses were performed with a 95% confidence interval (CI 95%), and results were considered statistically significant for *p*-values below 0.05.

## 3 Results

### 3.1 Influence of demographic characteristics

The results obtained from this study provide a significant insight into the current state of cheese culture in Morocco, particularly in terms of aged cheese. Although Morocco has not historically specialized in the production of this type of cheese, the data collected reveal an encouraging dynamic that testifies to the gradual integration of this product into local eating habits. This development is all the more notable given that mature cheese, in addition to its organoleptic qualities, is recognized for its scientifically proven nutritional value, due to its richness in protein, calcium, and essential vitamins. It appears essential to further enhance the value of this product, not only in terms of local production, but also through initiatives aimed at promoting its consumption among different sections of the population. The results indeed show that Moroccan consumers exhibit a significant interest in this type of cheese, but also that this interest is modulated by several determining factors. These include the socioeconomic and sociodemographic characteristics of the population studied, as illustrated in Table 1, which reveals that age, income level, region, and consumption status vary significantly by gender, with high significance (*p* < 0.01).

**Table 1:** Characteristics of the studied population.

Characteristics of the studied population		Gender						*P*-value
		Woman		Men		Total		
		*N*: 256	Subtable valid *N* %	N: 254	Subtable valid *N* %	*N*: 510	100%	
*Age*	18 to 39 years	185	36.3%	46	9.0%	231	45.3%	<0.01
	40 to 64 years	51	10.0%	188	36.9%	239	46.9%	
	>65 years	20	3.9%	20	3.9%	40	7.8%	
*Income level*	Very high	33	6.5%	70	13.7%	103	20.2%	<0.01
	High	191	37.5%	32	6.3%	223	43.7%	
	Low	20	3.9%	20	3.9%	40	7.8%	
	Moderate	12	2.4%	132	25.9%	144	28.2%	
*Frequency of matured cheese consumption*	Very frequently (daily)	12	2.4%	59	11.6%	71	13.9%	<0.01
	Frequently (several times a week)	30	5.9%	130	25.5%	160	31.4%	
	Occasionally (a few times a month)	177	34.7%	20	3.9%	197	38.6%	
	Rarely (fewer than two times a month)	37	7.3%	45	8.8%	82	16.1%	
*Regions of Morocco*	Béni Mellal Khénifra	11	2.2%	11	2.2%	22	4.3%	<0.01
	Casablanca Settat	51	10.0%	47	9.2%	98	19.2%	
	Dakhla Oued Ed Dahab	13	2.5%	8	1.6%	21	4.1%	
	Drâa Tafilalet	18	3.5%	10	2.0%	28	5.5%	
	Fès Meknès	20	3.9%	22	4.3%	42	8.2%	
	Guelmim Oued Noun	12	2.4%	8	1.6%	20	3.9%	
	Laayoune Sakia Al Hamra	6	1.2%	8	1.6%	14	2.7%	
	Marrakech Safi	27	5.3%	32	6.3%	59	11.6%	
	Oriental	15	2.9%	13	2.5%	28	5.5%	
	Rabat Salé Kénitra	34	6.7%	46	9.0%	80	15.7%	
	Souss Massa	23	4.5%	25	4.9%	48	9.4%	
	Tanger Tétouan Al Hoceïma	26	5.1%	24	4.7%	50	9.8%	

Results are based on nonempty rows and columns in each innermost subtable. The Chi-square statistic is significant at the 0.05 level.

#### 3.1.1 Gender influence on consumption

**Figure 1:**
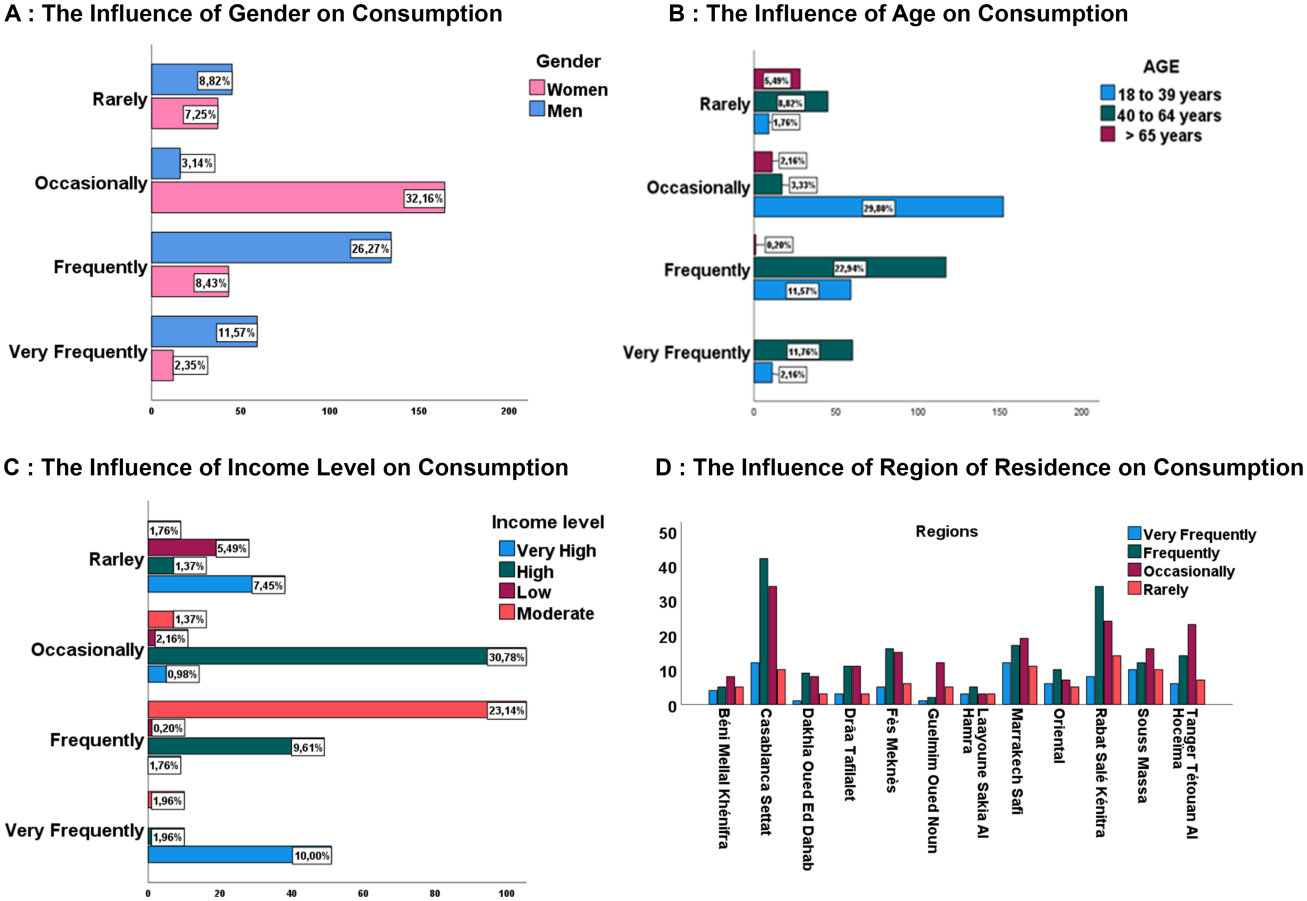
Socio-demographic factors influencing consumption. **(A)** The influence of gender on consumption. **(B)** The influence of age on consumption. **(C)** The influence of income level on consumption. **(D)** The influence of region of residence on consumption.

The chi-squared test revealed a significant association between the frequency of consumption of mature cheeses and gender, with a Cramer’s V correlation coefficient of 0.62 and a *p*-value below 0.001 (Table 2). Analysis of the results in Table 1 and Figure 1A indicates that consumption behavior differs significantly between men and women. Although the distribution between the sexes is relatively balanced, with 49.8% of men and 50.2% of women in the population studied, men seem to consume mature cheeses more frequently. Indeed, 37.8% of men reported regular consumption of these products, compared to only 10.8% of women. While occasional consumption, defined as consumption a few times a month, is significantly more widespread among women, with 32.2% of them adopting this consumption pattern, compared with just 3.1% of men. This disparity suggests that women prefer a more moderate or intermittent approach to consuming mature cheeses. Conversely, there is a slight variation in terms of infrequent consumption, with 7.3% of women and 8.8% of men reporting sporadic consumption of these products. A comparative analysis of trends reveals a heterogeneous distribution of consumption patterns. This suggests that men exhibit greater loyalty and more sustained consumption of mature cheeses, which contrasts with women’s habits, which tend toward more occasional consumption.

**Table 2:** Symmetric measures between sociodemographic variables.

Socio-demographic characteristics	Value phi	Chi-square tests	df	*p* value	Cramer’s V
	Frequency of aged cheese consumption				
Gender	0.627	200,363^a^	3	<0.001	0.627
Age	0.788	316,775a	6	<0.001	0.557
Income level	1.018	528,935^a^	9	<0.001	0.589
Region	0.257	33,806^a^	33	>0.05	0.149
Accessibility	0.807	332,510^a^	6	<0.001	0.571
Retail outlets	0.693	244,724^a^	8	<0.001	0.49
Brand	0.65	215,446a	3	<0.001	0.65
Origin	0.856	373,571^a^	6	<0.001	0.605
Labels	0.964	473,544^a^	3	<0.001	0.964
Purchasing power	0.237	28,714^a^	6	<0.001	0.168

^a^indicates statistical significance between the compared groups.

#### 3.1.2 Impact of age on consumption frequency

The statistical analysis also revealed a significant correlation between age and the frequency of consumption of mature cheeses, with a Cramer’s V value of 0.62 and a *p*-value below 0.001 (Table 2). Analysis of the results (Table 2) also highlights trends specific to each age group. Younger adults (18–39 years of age) and older adults (40–64 years of age) exhibit relatively similar levels of interest in mature cheeses, with rates of 45.3 and 46.9, respectively. In contrast, people aged 65 years and over represent a lower proportion of consumers interested in these products, at just 7.8%. Regarding consumption frequency, as shown in Figure 1B, young adults (18–39 years of age) stand out with a regular consumption pattern, representing 43.5% of loyal consumers among those who consume aged cheese, with 13.7% consuming it frequently and 29.8% occasionally. This group holds a comparable weight to that of older adults (40–64 years of age), who also exhibit frequent consumption at 34.7%, while 3.3% adopt a more moderate consumption pattern. In contrast, consumption among seniors (65 years and older) remains marginal, accounting for only 7.7%, with a predominance of rare consumers 5.5% and a complete absence in the frequent consumers category (Table 3).

**Table 3:** Discrimination measures.

	Dimension		Mean
	1	2	
Region	0.189	0.480	0.334
Label	0.892	0.004	0.448
Nutritional value	0.646	0.788	0.717
Quality	0.932	0.703	0.818
Cheese origin	0.712	0.274	0.493
Age	0.269	0.585	0.427
Accessibility	0.675	0.235	0.455
Price	0.915	0.658	0.787
Retail outlets	0.875	0.072	0.473
Gender	0.000	0.589	0.294
Income level	0.383	0.701	0.542
Frequency of consumption	0.942	0.817	0.879
Brand	0.574	0.000	0.287
Active total	8.004	5.906	6.955
% of variance	61.568	45.432	53.500

Model summary				
Dimension	Cronbach’s alpha	Variance accounted for		
		Total (eigenvalue)	Inertia	% of variance
1	0.948	8.004	0.616	61.568
2	0.900	5.906	0.454	45.432
Total		13.910	1.070	
Mean	0.928^a^	6.955	0.535	53.500

a Mean Cronbach’s alpha is based on the mean eigenvalue.

#### 3.1.3 Effect of income on consumption patterns

The effect of income on the consumption habits of mature cheeses is particularly striking, influencing both the frequency of purchase and the choice of products. A significant correlation was observed, with a Cramer’s V coefficient of 0.6 and a *p*-value below 0.001 (Table 2). According to the results presented in Table 1, high-income consumers are the group most interested in mature cheeses, accounting for 43.7%. This can be supplemented by those with very high incomes, who make up 20.2%, for a total of 63.9%. This group far exceeds consumers on moderate incomes (28.2%), while individuals on very low incomes show less interest in these products, with just 7.8%, reflecting their limited purchasing power. In terms of frequency of consumption, individuals with high and moderate incomes predominate in all categories. For frequent consumers, they represent 23.3 and 25.1%, respectively, while consumers on low and very low incomes are almost absent from this category, at 0.2%. A similar trend is observed for occasional consumption, where individuals on high incomes account for 31.8%, while those on low incomes begin to appear gradually at 2.2%. In the case of more infrequent consumption, the latter group reaches 5.5%, compared with 10.6% for financially well-off consumers. As illustrated in Figure 1C.

#### 3.1.4 Impact of place of residence on consumption

The impact of place of residence on the consumption of mature cheeses varies considerably between the different regions of Morocco. Large urban areas, characterized by high urbanization, population density, and dynamic economic activity, demonstrate a heightened interest in these products. However, this variable does not exhibit an objective association with cheese consumption, showing less significance (*p* > 0.05), was analyzed by the chi-squared test, and a Cramer’s V coefficient of 0.2 (Table 2; Figure 1D). Analysis of the results reveals that regions with a well-developed infrastructure and a diversified commercial offer are the most inclined to consume mature cheeses, in contrast to less urbanized areas, where the population is more dispersed and access to specialized products is more limited. Residents of the Casablanca-Settat region are the primary regular consumers, with a rate of 19.2%, followed by those of Rabat-Salé-Kénitra at 15.7%, Marrakech-Safi at 11.6%, and Tangier-Tétouan-Al Hoceima at 9.8%. These regions form the core of loyal consumers of mature cheeses. At an intermediate level, the Fès-Meknès region has a consumption rate of 8.2%, while Laâyoune-Sakia El Hamra has the lowest proportion, at just 2.7%. A similar trend is observed for occasional and infrequent consumers, as illustrated in Figure 1D.

### 3.2 Factors influencing access to mature cheeses and their impact on consumption

The factors that affect access to mature cheeses play an essential role in their consumption. The data collected indicate that this accessibility is strongly influenced by the availability of specialist outlets, which are still restricted in some regions, thus limiting access for part of the population. This part aims to assess the impact of locality of residence, sales outlets, and income levels on access to mature cheeses, and consequently on consumption frequency.

#### 3.2.1 Accessibility to mature cheeses

The consumption of mature cheeses in Morocco is intrinsically linked to their accessibility, which depends on various factors, including geographical distribution and the diversity of sales outlets. The results of this study reveal a significant dependency between the frequency of consumption and the accessibility of mature cheeses, as validated by a chi-squared test with a *p*-value below 0.001 and a Cramer’s V coefficient of 0.6 (Table 2).

**Figure 2:**
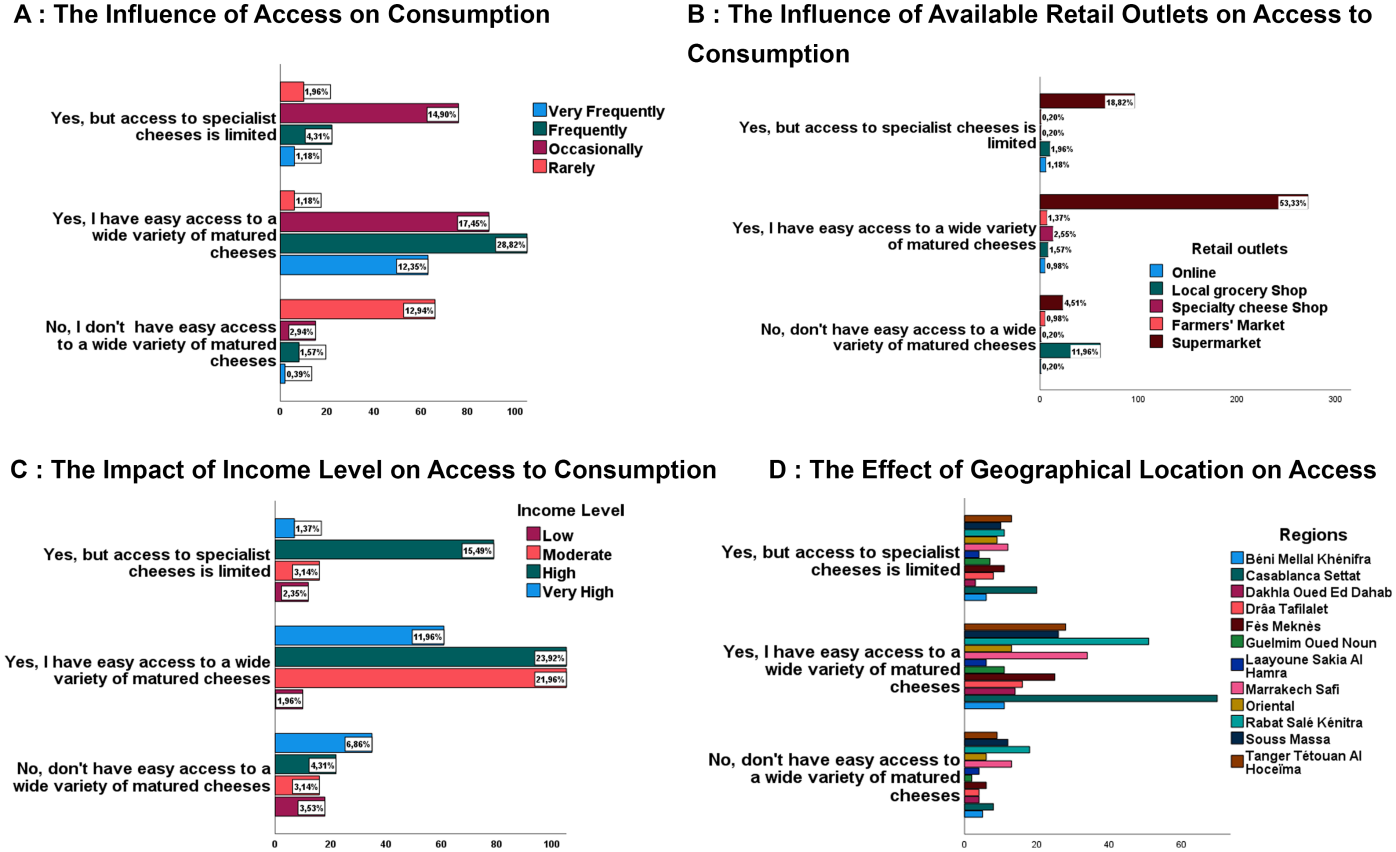
Determinants of access to aged cheeses. **(A)** The influence of access on Consumption. **(B)** The Influence of available retail outlets on access to consumption **(C)** The impact of income level on access to consumption. **(D)** The effect of geographical location on access.

The data analysis, illustrated in Figure 2A, shows that frequent to widespread consumption is mainly observed among people with easy access to a wide variety of specialty cheeses, representing 41.1% of respondents. By contrast, this rate drops to 5.49% among people with limited access. Similarly, occasional consumption accounted for 17.5% of those with better access, compared with 14.9% of those with restricted access. The impact of accessibility is also reflected in the infrequent consumption of mature cheeses. Indeed, only 1.2% of individuals with easy access rarely consumed these products, compared with 1.9% of those with limited access. Moreover, among people with access difficulties, the proportion of occasional and frequent consumers remains low, at 2.9 and 1.9% respectively, while the proportion of infrequent consumers rises to 12.9%. These results suggest that the limited consumption of mature cheeses is influenced by several factors, with accessibility playing a decisive role.

#### 3.2.2 Regional influence on accessibility

The regional influence on accessibility to aged cheeses is reflected in geographical disparities linked to distribution and commercial infrastructure, with moderate significance (*p* < 0.05) and a Cramer V coefficient of 0.2 (Table 2). The results presented in Figure 2B indicate that highly urbanized regions, such as Casablanca-Settat and Rabat-Salé-Kénitra, followed by Marrakech-Safi and Tangiers-Tétouan-Al Hoceïma, dominate among those whose residents claim to have wide and easy access to a wide variety of mature cheeses. A similar trend is observed for those reporting limited access, although with a slight regional repositioning, where Tangier-Tétouan-Al Hoceïma is ahead of Rabat-Salé-Kénitra. In contrast, those reporting no access, generally located in smaller, less urbanized towns or rural areas, represent a smaller proportion, with a predominance of residents of Rabat-Salé-Kénitra, followed by Marrakech-Safi, Souss-Massa, Tangier-Tétouan-Al Hoceïma, and Casablanca-Settat, whereas the other regions present a relatively even spread, as illustrated in Figure 2B. Moreover, Laâyoune-Sakia El Hamra has a low representation in all accessibility categories, which corroborates the trends observed in Table 1.

#### 3.2.3 Accessibility and retail outlets

As illustrated in Figure 2B, the accessibility of mature cheeses is closely correlated with regional disparities, influenced by various economic factors specific to each region. Among these factors, the availability and diversity of sales outlets play a significant role, as confirmed by a significant association (*p* < 0.001) and a V Cramer coefficient of 0.5 (Table 2). The data in Figure 2C reveal that 59.8% of respondents stated they had easy access to a wide variety of aged cheeses, with a marked predominance of supermarkets as the main supply channel 53.3%, or more than half the cases. Therefore, these consumers benefit from a diversified range of specialty cheeses. Conversely, among the 21.4% of individuals reporting limited access, of whom 18.8% indicated restricted accessibility, supermarkets remain the dominant option. In comparison, specialist shops and local grocery shops account for only between 1 and 2% of choices, reflecting an under-exploitation of these distribution channels. Finally, 17.9% of respondents reported having no direct access to mature cheeses, but were seeking alternatives such as purchasing online. However, in this case, purchasing through local grocery outlets is preferred 11.9%, compared to only 4.5% who turn to supermarkets.

#### 3.2.4 Accessibility and income level

Figure 2D shows a significant positive correlation between income level and access to mature cheeses, with a *p*-value below 0.001 and a Cramer’s V coefficient of 0.3 (Table 2). Respondents with very high incomes (11.9%) and high incomes (23.9%), collectively accounting for 33.9% of the sample, largely dominate the category with easy access to a wide variety of cheeses, unlike respondents with moderate incomes (21.9%) and low incomes, 1.9%, who are significantly less represented. Furthermore, among individuals reporting limited access, those with high to very high incomes continued to predominate, with a total rate of 16.9%. This was followed by those with moderate incomes, at 3.1%, and those with low incomes (2.4%). In addition, the “no easy access” category exhibits an over-representation of respondents with high incomes (11.2%), moderate incomes (3.3%), and low incomes (3.5%).

### 3.3 Impact of choice criteria on the consumption of mature cheeses

The survey reveals that purchasing behavior and product acceptability are related to a combination of properties considered primary drivers of purchase, specifically nutritional value, quality, and origin of the product. However, differences of interest emerge regarding the importance attributed to certain characteristics, with some respondents judging them to be more important than others.

#### 3.3.1 Decisive factors in the purchase

**Figure 3:**
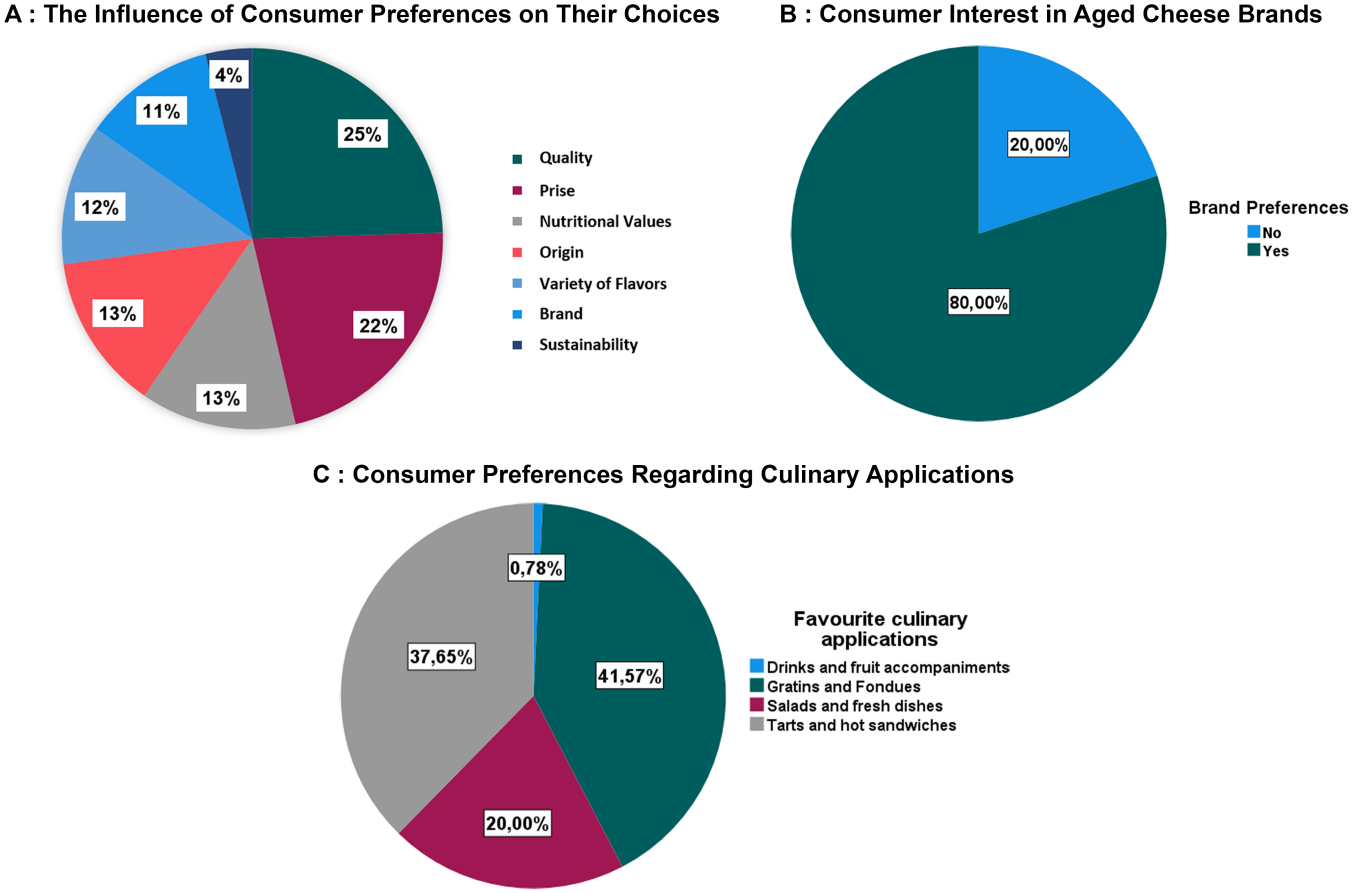
Criteria for choosing aged cheese. **(A)** The influence of consumer preferences on their choices. **(B)** Consumer interest in aged cheese brands. **(C)** Consumer preferences regarding culinary applications.

The results illustrated in Figure 3A, presented in graphical form, highlight the determining characteristics in the purchase of mature cheeses, which vary according to consumer preferences and priorities. The analysis reveals that quality is the main criterion, cited by 23% of respondents as an absolute priority, slightly surpassing the price, at 20%. Next come the nutritional value and origin of the product, each accounting for 12% of the preferences expressed. Moreover, the search for a variety of flavors concerned 11% of consumers, playing an essential role in the initial appeal of the product, particularly for those less familiar with mature cheeses. The brand is also a decisive factor for 10% of respondents. At the same time, other criteria, although less influential, contribute to the purchasing decision, notably durability and recommendations from friends and family, each accounting for 4%.

#### 3.3.2 Influence of local culture and culinary practices

Regarding the culinary uses of mature cheeses, the results are presented in graphical form in Figure 3C, as they relate to preferences with no significant dependence on consumption frequency (*p* > 0.05), indicating that they do not have a direct influence on consumption but rather are related to consumer choice. The data shows that gratins and fondues account for 41.6% of declared consumption, followed by tarts and hot sandwiches with 37.7%, while salads and fresh dishes are preferred by 20% of respondents. A very small proportion, 0.8%, reported using mature cheeses with drinks and fruit.

#### 3.3.3 Brand importance in the purchasing decision

The results presented in Figure 3B, regarding the impact of brand as a marketing characteristic facilitating acceptability, reveal a moderate level of interest among Moroccan consumers in branded aged cheeses. These findings support the analysis of selection factors, with brand maintenance as one of the key elements influencing purchasing decisions for a segment of the population. Specifically, 80% of respondents indicate an interest in brands or express a preference for specific ones, while 20% do not consider branding a relevant criterion. These data highlight the significance attributed to this factor by Moroccan consumers, although other criteria appear to be more decisive, as illustrated in Figure 3A. This observation explains why branding did not demonstrate a significant impact on consumption; a conclusion further supported by the chi-squared test.

### 3.4 Impact of economic factors on consumption

The results confirmed that economic factors play a decisive role in the consumption of mature cheeses, influencing both consumers’ purchasing power and their eating habits. For example, price is a significant barrier, particularly for consumers on low and/or average incomes, limiting their access to these products. Furthermore, the origin of the cheeses proved to be a predominant choice criterion. Moreover, the results highlight the population’s sensitivity to signs of quality, such as labels, as well as their preferences regarding innovations in this sector.

#### 3.4.1 Influence of product origin

**Figure 4:**
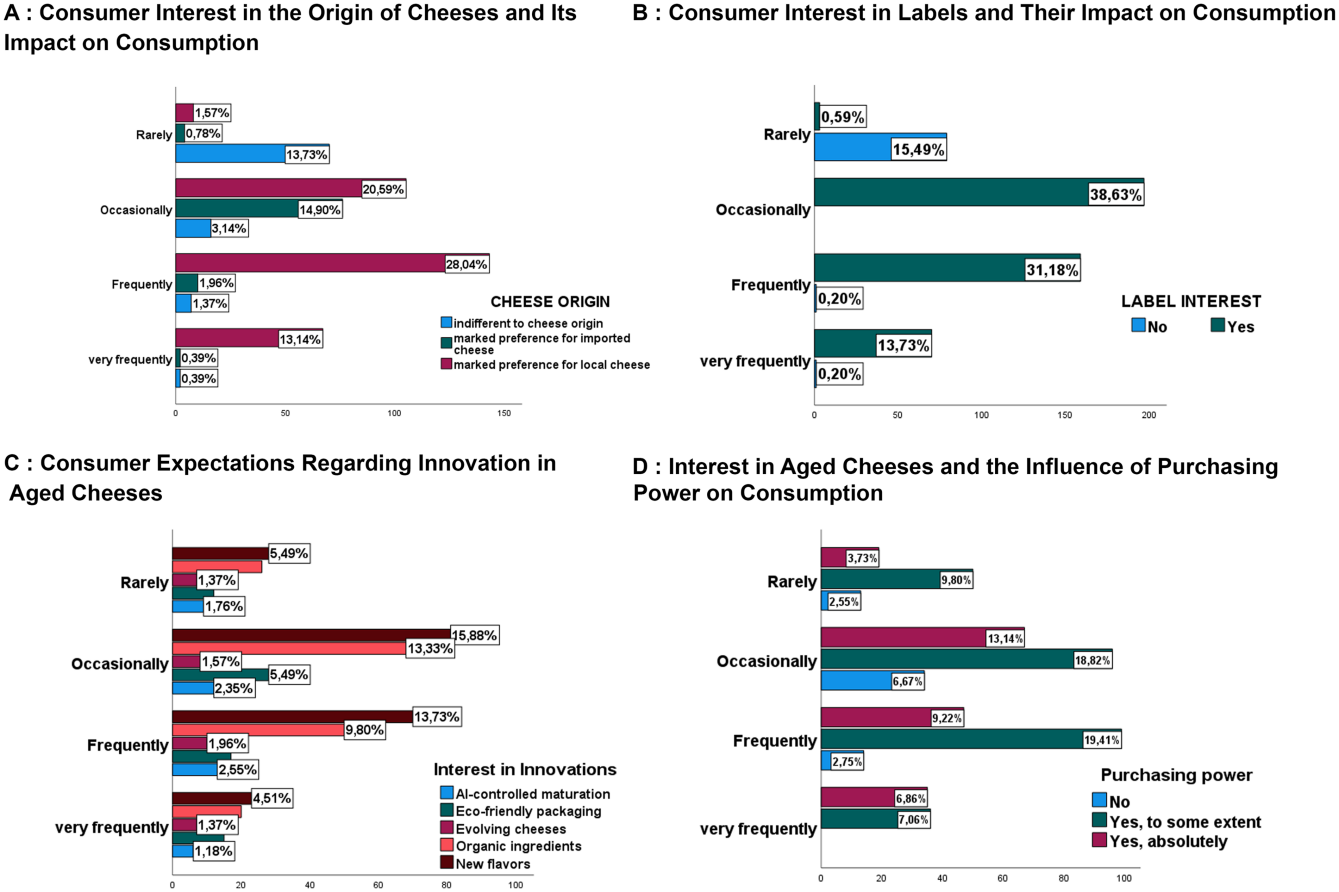
Economic factors influencing the consumption of aged cheese. **(A)** Consumer interest in the origin of cheeses and its impact on consumption. **(B)** Consumer interest in labels and their impact on consumption. **(C)** Consumer expectations regarding innovation in aged cheeses. **(D)** Interest in aged cheeses and the influence of purchasing power on consumption.

As shown in Figure 4A, frequency of consumption is closely linked to the origin of the aged cheese, indicating a marked preference for local products, confirmed by the chi-squared test’s statistics revealing a *p*-value below 0.001 and a Cramer’s V coefficient 0.6 (Table 2), which showed a significant relationship between these variables. Frequent and very frequent consumers exhibit a total preference for local cheeses of 43.3%, while only 3.3% prefer imported cheeses, compared to an almost negligible share of 0.4% for those with no preference at all. The same trend is apparent among occasional consumers, with 18.4% opting for local cheeses, compared to 13.9% for imported cheeses and 2.9% for those with no preference. Conversely, infrequent consumers are characterized by a high level of indifference toward the origin of the product, with 18.4% showing no preference. Only 1.6% preferred local cheeses and 0.8% imported products, reflecting a low level of product knowledge and interest in their characteristics.

#### 3.4.2 Moroccan consumers’ interest in labels

The results of this study also show that Moroccan consumers’ interest in labels reflects an increased search for quality and confidence in their food choices, particularly for mature cheeses. This influence is highly significant in terms of consumption frequency, as confirmed by the chi-squared test, with a *p*-value below 0.001 and a Cramer V coefficient of 0.9 (Table 2). According to the results illustrated in Figure 4B, the majority of loyal consumers of aged cheeses (both frequent and occasional consumers) pay particular attention to labels. Indeed, 84.1% of the population studied showed a marked interest in these signs of quality. This trend is reflected in the different consumption categories: among those who consume frequently or very frequently, 48.2% show a strong attachment to the labels, against only 0.4% who show no interest at all. In contrast, occasional consumers show a strong attachment to labels, with a complete absence of respondents who consider them useless. On the other hand, among infrequent consumers, a higher proportion (15.5%) are indifferent toward labels, compared to just 0.6% who are interested.

#### 3.4.3 Preferences of Moroccan consumers regarding innovation

The interest of Moroccan consumers in innovation plays a decisive role in the evolution of the aged cheese market. This interest was assessed as an individual preference, and since it is specific to each consumer, it does not show a significant correlation with consumption frequency (*p* > 0.05). However, the results illustrated in Figure 4C reveal a consistent trend across all consumption categories (very frequent, frequent, occasional, and rare), highlighting a predominance of consumers who favor innovation in terms of flavor diversification, representing a total of 39.6% of the study population. Similarly, 32.2% of respondents attach particular importance to the use of organic ingredients, followed by 14.1% who show sensitivity to environmental concerns. Furthermore, 7.8% of consumers express an interest in integrating artificial intelligence into the manufacturing process, particularly for controlling ripening, while the smallest proportion, 6.3%, are those who encourage the production of evolving cheeses, that is, cheeses that continue to ripen after marketing.

#### 3.4.4 Purchasing power of the Moroccan population

The purchasing power of the Moroccan population exerts a direct influence on the consumption of aged cheeses. This influence was confirmed as significant by the chi-squared test, with a *p*-value below 0.001 (Table 2). The results presented in Figure 4D reveal that only 11.9% of the study population (made up of 2.9% frequent consumers, 6.5% occasional consumers and 2.5% infrequent consumers) believe that their purchasing power limits their frequency of purchase of mature cheeses, suggesting a preference for more affordable products in terms of price. It is noteworthy that no consumer in the “very frequent” category has this limitation, which reflects their interest in mature cheeses. In contrast, 35.9% of respondents expressed their willingness to pay for high-quality aged cheeses, with 17.6% of them being regular consumers. More than half of the survey population (55.1%) is also willing to pay a high price, but this willingness is contingent upon other factors, indicating that price remains a crucial criterion in product choice, as shown in Figure 3A.

## 4 Discussion

This study analyzed the various factors influencing the purchasing decisions and behavior of Moroccan consumers of aged cheese, highlighting the interrelationships between these variables. By conducting a multiple correspondence analysis, it was possible to identify underlying structures within the data and group the factors correlated with each other, thereby making it possible to identify standard dimensions that significantly influence consumers’ choice. The results confirm a general trend toward growth in the consumption of aged cheeses in Morocco (17). However, although this development is evident, consumers’ level of knowledge about the specific characteristics of these products remains moderate. This situation is not isolated, as it reflects a dynamic observed on an international scale, where cheese culture is primarily evolving from a consumer perspective, while local production remains limited (6).

**Figure 5:**
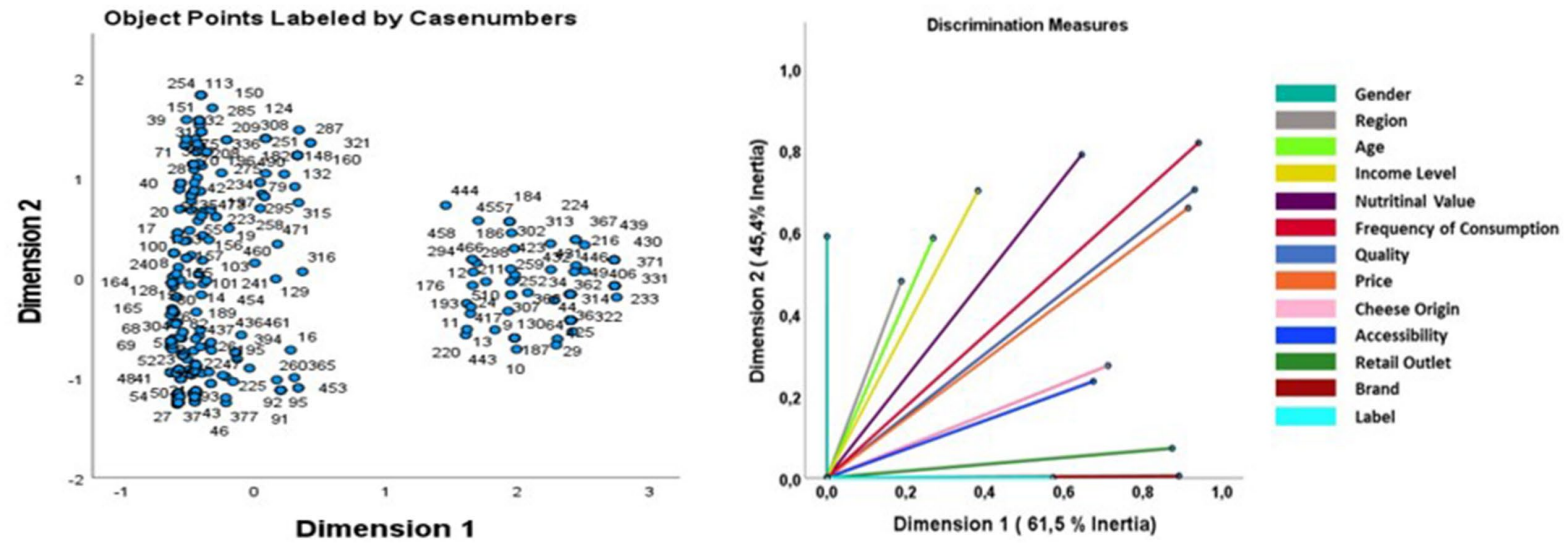
Analysis of associations between influencing factors using multiple correspondence analysis (MCA).

Principal component analysis identified three main categories of variables that significantly influence consumer purchasing behavior. As illustrated in Figure 5, these variables are organized around two main dimensions.

The first dimension, with a value of 8.004, groups the economic factors, which encompass the four pillars of the marketing mix, namely, product, price, place, and promotion, and their recognized importance (38). These factors are divided into two distinct sub-groups. The first highlights the frequency of consumption about the decisive criteria, particularly quality, which is positioned as a priority element, surpassing both price and nutritional value, as confirmed by the results presented in Figure 3A. This observation highlights the rationale of the quality/price ratio desired to guarantee the acceptability of the product (39), particularly within a context where aged cheeses constitute an emerging category in the Moroccan market. The second subgroup relates to the intrinsic characteristics of the product that can be exploited for marketing purposes, such as the origin of the product, which exhibits a significant correlation with consumption frequency, as well as access methods, points of sale and quality signs (labels), as illustrated in the results section of Figure 4B.

These findings suggest that the Moroccan market offers favorable potential for the development of mature cheese production. The results obtained align with the existing literature on the growth in consumption of these products, highlighting the increasing interest in local food products (40) and underscoring the relationship between supply and demand (15), which could constitute a lever for investors wishing to become engaged in this sector. The study highlights a strategic opportunity for economic actors seeking to invest in an industry characterized by relatively little local competition, while increased demand for local products is being observed. This trend can be explained by Moroccan consumers’ preoccupation with sustainability (26), as well as their perception of freshness as a quality indicator that directly influences their purchasing decisions (41). Additionally, freshness has an impact on price, potentially reducing it lower import costs (42). The value of this characteristic could be enhanced by obtaining a quality label (43), particularly since the Moroccan population is already aware of the signs of certification, as shown in Figure 4, and is thus less likely to be driven by innovation. Given that this product segment attracts consumers seeking distinctive and unique characteristics, as opposed to mass-produced and standardized industrial cheeses (44), which is a significant asset in a context where the cheese culture is still emerging. Such a strategy could enable local producers to stand out from the crowd and position themselves effectively in a market perceived as saturated by imported products, often associated with recognized authenticity (45). Although this concept can also be applied in Morocco, where it is rooted in a distinct terroir, artisanal expertise, and traditional recipes that ensure identity and uniqueness, there is significant potential for labeling (21). Moreover, this type of production to revitalize the rural economy and foster local tourism, provided it is supported by stronger regional collaboration and appropriate institutional backing (46). Moreover, the availability of mature cheeses and the diversity of sales outlets play a crucial role in optimizing consumption status, as demonstrated by the findings presented in Figure 2B. These factors directly influence accessibility and, consequently, consumption. It is therefore essential to optimize distribution by targeting preferred points of sale, particularly local grocery shops, to facilitate access for isolated populations and improve consumption status (47). In summary, the results suggest that a quality product, offered at an affordable price, recognized nutritional value, and supported by an effective communication strategy focused on accessibility and availability across various points of sale, could differentiate itself on the national market. The use of labels, particularly “local,” could further reinforce this differentiation.

The second dimension, with a value of 5.906, highlights the influence of socioeconomic factors on the consumption of mature cheeses. The primary determining factor is income level, which indirectly reflects purchasing power and conditions accessibility to these products (48). The study emphasizes the necessity of tailoring the offer to each income segment, particularly by diversifying the pricing range while maintaining an acceptable standard of quality to promote more inclusive consumption. This approach is especially essential for low-income consumers, for whom rising food prices can exacerbate precariousness and limit access to beneficial products (49). However, the results reveal that even a proportion of low-income respondents are willing to pay a high price for local cheese, which the patriotism and ethnocentrism could explain. These factors play a significant moderating role in purchasing decisions, directing consumer preference toward local products to the detriment of imported alternatives (50). This differentiates consumers from true enthusiasts, for whom price is less important (44). Similarly, age is a factor that moderately influences purchasing behavior (51). In this respect, the results indicate that the 18–39-year age group is more inclined to discover new products (52) and to diversify their eating habits, particularly under the influence of international trends and an increased search for novelty. In contrast, consumers aged 40 years and above showed less frequent consumption of mature cheeses, with the main reasons cited for this being to reduce fat or cholesterol (53), particularly as awareness campaigns tend to discourage the consumption of high-fat products (54). This observation underscores the importance of an effective communication strategy, which highlights the nutritional benefits of mature cheeses to counter certain preconceived ideas and encourage their adoption by all categories of consumers (55). The regional influence is also significant, both in terms of consumption and accessibility (56). A notable disparity is observed between urban and rural areas, with the former benefiting from easier access to a diversified offer. Conversely, consumers in less urban areas are more constrained by accessibility barriers and local cultural preferences. This highlights the importance of an effective strategy to promote fairer access to mature cheeses.

Finally, gender also plays a moderate role in consumption patterns (57). Nutritional and behavioral factors may explain the differences observed between men and women. Given their higher energy requirements and greater physical activity, men are more inclined to consume nutrient-rich products such as mature cheeses. Conversely, women, particularly in urban areas, seem to prefer these products in specific contexts (58), often linked to special occasions or a quest for culinary refinement. In addition, women are more likely than men to express concern about the broader impacts of consumption and to adjust their behavior accordingly, which limits the frequency of their consumption (59). These observations suggest that marketing and communication strategies should be tailored to different age groups and gender preferences to optimize the acceptance of mature cheeses (60). Other aspects related to individual cultural preferences have been analyzed, highlighting differences in the culinary applications and common uses of mature cheeses (61); however, these factors did not demonstrate a significant correlation with consumption. Furthermore, geographical and commercial accessibility, highlighted by the two dimensions, constitutes a strategic lever that should not be overlooked. It seems essential to adapt distribution and marketing policies according to consumers’ place of residence, by diversifying sales outlets and ensuring a greater presence across the entire country through sales intermediaries (62), while limiting the need for consumers to look for these products outside their usual sales outlets (63). Given that spatial and social constraints influence food purchases, the choice of outlet is determined by physical accessibility, whereas the selection of products is based on the perception of value, price, and quality (47). This approach would encourage more balanced consumption and expand the base of regular consumers.

This study demonstrates that the Moroccan population manifests a growing interest in aged cheeses, in line with international consumption trends. However, this culture is still emerging in Morocco, which represents an opportunity for local producers to invest in the sector, taking into account the findings of this research to meet consumer expectations better and structure an offer adapted to the specific characteristics of the national market.

## 5 Conclusion

The consumption of mature cheeses is emerging as a significant trend in Morocco, despite the practice not being historically rooted in the country’s food culture. This trend is underpinned by several factors, including rising living standards, openness to external cultural influences, and recognition of the nutritional benefits of mature cheeses. However, this dynamic has not been accompanied by a proportional increase in local production, leaving the country dependent on imports. The results of this study indicate that the consumption of mature cheeses is influenced by socioeconomic factors, particularly the price-quality ratio, with a marked preference for product quality rather than price, as well as a growing interest in quality labels and the accessibility of sales outlets. These factors showed a significant correlation with the frequency of consumption. Furthermore, approximately 63.3% of respondents prefer local products, a crucial factor in assessing the success of this sector. This finding is particularly motivating, especially when combined with the large proportion of 32.9% of so-called amateur consumers who are willing to pay a high price for aged cheeses, unconditionally. Although knowledge of aged cheeses, both in terms of innovation and specific characteristics, remains moderate, it is constantly improving, testifying to a gradual evolution in cheese culture in Morocco. Consumers are gradually becoming more aware of the reasons for revaluing these products, despite their absence in the local culinary tradition. However, although consumers are willing to embrace quality cheeses and acceptability is assured, accessibility to these products remains a challenge due to the lack of affordable local alternatives, underlining the necessity to strengthen local supply and further promote Moroccan products by adapting them to local preferences and these observed trends, to reduce dependence on imports.

### 5.1 Limitation

Although this study provides a valuable insight into consumption trends for mature cheeses in Morocco, it could be enhanced by a more in-depth exploration of specific dimensions. An analysis of the influence of sensory marketing and the perception of quality labels and certifications, particularly as a function of consumers’ level of awareness, would offer additional perspectives. It is essential to acknowledge that the chosen online distribution method may have influenced the demographic composition of respondents. Indeed, since older individuals are often less active online and rural populations tend to have limited exposure to modern consumption patterns, the online distribution method did not guarantee a completely balanced demographic representation. These methodological limitations should be carefully considered in future studies aiming for greater population representativeness. Additionally, the questionnaire-based approach relies on self-reporting by participants, which may introduce certain biases, such as social desirability bias or memory recall errors. However, a complementary qualitative approach, such as interviews or focus groups, would allow for a deeper exploration of consumers’ motivations and perceptions of mature cheeses, as well as the influence of foreign cultural references on the Moroccan market. Such an approach would not only provide a more nuanced understanding of preferences. Still, it would also address key issues, such as raising awareness of the nutritional benefits of mature cheeses and challenging certain preconceived notions. In addition, directly involving producers in the analysis of this market would be an interesting way to identify their concerns and gain a better understanding of the obstacles to the sector’s development. The use of analysis tools tailored to the interviews would also broaden the scope of this study and guide the stakeholders toward strategies better adapted to the expectations and dynamics of the Moroccan market.

## Data Availability

The raw data supporting the conclusions of this article will be made available by the authors, without undue reservation.
