# Supplementary material for: Determinants of aged cheese consumer preferences in Morocco—a cross-sectional study of economic, cultural, and social factors influencing purchasing behaviors
Source: Front Nutr. 2025 Jul 11;12:1600873. doi: 10.3389/fnut.2025.1600873 (PMC12289578; doi:10.3389/fnut.2025.1600873)
Supplement: Supplementary file 1 [file Data_Sheet_1.pdf]

Assessment of Aged Cheese Consumer Preferences in Morocco: Your Response, Your Signature for Cheese Innovation

The primary objective of this study is to understand consumer preferences, with a particular focus on Aged Cheeses, in Morocco. This research aims to analyze the sensory, nutritional, and environmental characteristics that influence consumer choices. It also examines purchasing behaviors and concerns related to product quality and sustainability.

The results of this survey will have a direct application in improving cheese production methods, with the goal of better meeting the needs and expectations of consumers who are mindful of quality, health, and the environmental impact of food products. Your response will help shape cheese innovation in Morocco.

Thank you for your participation and your contribution to the continuous improvement of the cheese industry in Morocco.

Note: Your honest opinion is highly valuable to us.

1. **Please specify your gender:**

Male

Female

2. **Please specify your age range:**

18 and over

40 and over

70 and over

3. **Please indicate your current income level:**

Very Low

Low

Moderate

High

Very High

4. **How often do you consume Aged Cheeses?**

Very Frequently (daily)

Frequently (several times a week)

Occasionally (once to a few times per month)

Rarely (less than once a month)

5. **Do you have easy access to a variety of cheese types, including specialty cheeses, where you currently live?**

Yes, I have easy access to a wide variety of specialty cheeses

Yes, but access to specialty cheeses is limited

No, I do not have easy access to a variety of cheeses, including specialty cheeses.

6. **In which locality do you reside?**
7. **Where do you usually purchase Aged Cheese?**

Supermarket

Local grocery store

Farmers' market

Specialty cheese shop

Online

Other

If other, please specify:

8. **What factors are most important to you when choosing Aged Cheese?**

Price

Quality

Origin (local or foreign)

Brand

Nutritional values

Variety of flavors

Organic certifications

Production conditions

Sustainability

9. **Among the following types of cheese, which do you prefer the most?**

Cooked pressed cheese

Uncooked pressed cheese

Soft cheese with washed rind

Soft cheese with bloomy rind

Blue-veined cheese

10. **Do you have preferred brands of Aged Cheese?**

Yes

No

If yes, please specify the brands of Aged Cheese you prefer:

11. **Do you prefer cheeses made with organic and natural ingredients?**

Yes

Absolutely Yes

To some extent, No

12. **Do you think that more detailed information about the ingredients (such as the origin of the milk and coagulant enzyme, or the production methods) would influence your purchasing decision?**

Yes

Strongly Yes

To some extent, No

13. **Do you prefer cheeses made from cow's milk, goat's milk, sheep's milk, or other milk sources?**

Cow's milk

Goat's milk

Sheep's milk

Other sources:

14. **How much do nutritional information (such as nutritional values, fat content, etc.) influence your cheese choice?**

Strongly

Moderately

No influence

15. **Are you concerned about the presence of additives or preservatives in the cheeses you purchase?**

Yes

Highly concerned

Yes, to some extent

No, not concerned at all

16. **Do you prefer cheeses with reduced fat content, or do you opt for cheeses rich in fat?**

Low fat content

Moderate fat content

High fat content

No preference

17. **Do you follow a specific diet (vegetarian, vegan, gluten-free, etc.) that affects your choice of Aged Cheese?**

Yes

No

If yes, please specify the diet you follow:

18. **Would you be willing to try Aged Cheeses made with unusual or exotic ingredients (e.g., spices, herbs, fruits)?**

Yes, ready to try

Yes, open to the experience

No, not interested

No, not feasible

19. **What texture characteristic do you specifically look for when evaluating an Aged Cheese?**

Elastic

Melty

Creamy

Granular

20. **What specific aromatic characteristic do you look for when evaluating an Aged Cheese?**

Fruity

Smoky

Herbal

Earthy

21. **What are the common culinary applications you prefer for Aged Cheeses, considering their specific aromatic profiles?**

Gratin dishes and Fondues

Pies and hot sandwiches

Salads and Fresh Dishes

Accompaniment for Drinks

Fruits

22. **Do you prefer to buy locally produced cheese, or are you open to imported cheese?**

Strong preference for local cheese

Slight preference for local cheese

No preference (open to imported cheese)

Slight preference for imported cheese

Strong preference for imported cheese

What are the reasons that influence your choice?

23. **Are you concerned about the environmental impact of the cheese industry?**

Yes, highly concerned

Yes, to some extent

No, not concerned at all

24. **What types of innovations would you like to see in Aged Cheeses in terms of Quality, Composition, or Flavor?**

AI-controlled aging

New flavors

Organic ingredients

Eco-friendly packaging

Evolving cheeses (that can be aged further at home)

Other

25. **Are you willing to pay a higher price for Aged Cheese made in an Organic and Sustainable way?**

Yes, absolutely

Yes, to some extent

No

26. **If you have any suggestions, ideas, or additional comments that you would like to share to enrich our understanding of consumer expectations or to improve your experience with aged cheeses, please provide them below.**
